# Discovery and Validation of Traditional Chinese and Western Medicine Combination Antirheumatoid Arthritis Drugs Based on Machine Learning (Random Forest Model)

**DOI:** 10.1155/2023/6086388

**Published:** 2023-02-15

**Authors:** Jijia Sun, Qinghua Ni, Fengyan Jiang, Baocheng Liu, Jianying Wang, Lei Zhang, Jihan Huang

**Affiliations:** ^1^Shanghai Collaborative Innovation Center of Traditional Chinese Medicine Health Service, Shanghai University of Traditional Chinese Medicine, Shanghai 201203, China; ^2^School of Pharmacy, Shanghai University of Traditional Chinese Medicine, Shanghai 201203, China; ^3^Center for Drug Clinical Research, Institute of Interdisciplinary Integrative Medicine Research, Shanghai University of Traditional Chinese Medicine, Shanghai 201203, China

## Abstract

The combination of traditional Chinese medicine (TCM) and Western medicine is a promising method for treating rheumatoid arthritis (RA). Combining the two fully exploits the advantages of Western and TCM to treat RA and has the potential to greatly improve the therapeutic effect on RA. In this study, we developed a combination drug training set by using 16 characteristic variables based on the characteristics of small molecules of TCM ingredients and Food and Drug Administration-certified combination drug data downloaded from the DrugCombDB database. Furthermore, we compared the prediction and classification abilities of five models: the *k*-nearest neighbors, naive Bayes, support vector machine, random forest, and AdaBoost algorithms. The random forest model was selected as the classification and prediction model for Western and TCM and Western combination drugs. We collected data for 41 small molecules of TCM ingredients from the Traditional Chinese Medicine Systems Pharmacology database and 10 small molecule drugs commonly used in anti-RA treatment from the DrugBank database. Combinations of Western and TCM for anti-RA treatment were screened. Finally, the CellTiter-Glo method was used to determine the synergy of these combinations, and the 15 most predicted drug combinations were carried out experimental verification. Myricetin, rhein, nobiletin, and fisetin had high synergy with celecoxib, and rhein had high synergy with hydroxychloroquine. The preliminary findings of this study can be further applied for practical clinical anti-RA combined treatment strategies and serve as a reference for clinical treatment of RA with integrated Western and TCM.

## 1. Introduction

Modern medical research has demonstrated that many diseases often present complex pathogenic mechanisms, and consequently, the past methods for treating diseases with drugs are no longer adequate. In recent years, drug combination has been reported to offer clear advantages in treating tumors, AIDS, cardiovascular diseases, osteoarthritis, and other complex diseases; it has been widely implemented in clinical practice [1–4], with treatment methods based on combined drugs demonstrating advantageous synergistic effects, or a “1 + 1 ≥ 2” effect. The therapeutic effects of two or more drug combinations are greater than those achieved when each drug is used alone. Therefore, developing and evaluating potentially synergetic combination drugs, whether for development of new drugs or clinical treatment optimization, are essential. However, most existing combination drugs have been developed on the basis of limited clinical experience or experimental strategies. Verified combination drugs with synergistic therapeutic effects currently comprise a small portion of listed drugs. Therefore, making full use of knowledge of existing drug combinations, discovering new drug combinations based on well-accepted and understood drugs, and establishing a model for evaluating the synergistic effects of drug combinations are key concerns that must be addressed.

Rheumatoid arthritis (RA) is a chronic, systemic, inflammatory disease of the synovial and other joints. RA is a chronic progressive autoimmune disease [5], and its underlying pathogenesis is currently unclear. However, studies have reported that the incidence of RA increases with age due to infection and inflammatory mediators, and approximately 0.3%–1.0% of the global population is affected by the disease every year [6, 7]. At present, the drugs used in clinical treatment of RA are mainly nonsteroidal anti-inflammatory drugs (NSAIDs), disease modifying antirheumatic drugs (DMARDs), and biological macromolecular therapy. Because RA is complex, several studies have explored different combination therapy strategies for its treatment [8–10]. However, some therapeutic drugs, such as methotrexate, may produce unfavorable side effects [11, 12]. Therefore, if sole use of anti-RA combination therapy has the potential to produce unfavorable side effects, long-term use in patients with RA may do greater harm. Traditional Chinese medicine (TCM) has long been used to treat RA, and several studies have found that combined anti-RA TCM and a compound prescription have not only anti-inflammatory, analgesic, immune regulation, and multilevel and multilink therapeutic effects but also the advantages of high safety, few adverse reactions, and low cost. Such a combination has gained attention in studies of RA treatment and has increasingly attracted international attention [13, 14].

**Figure 1:**
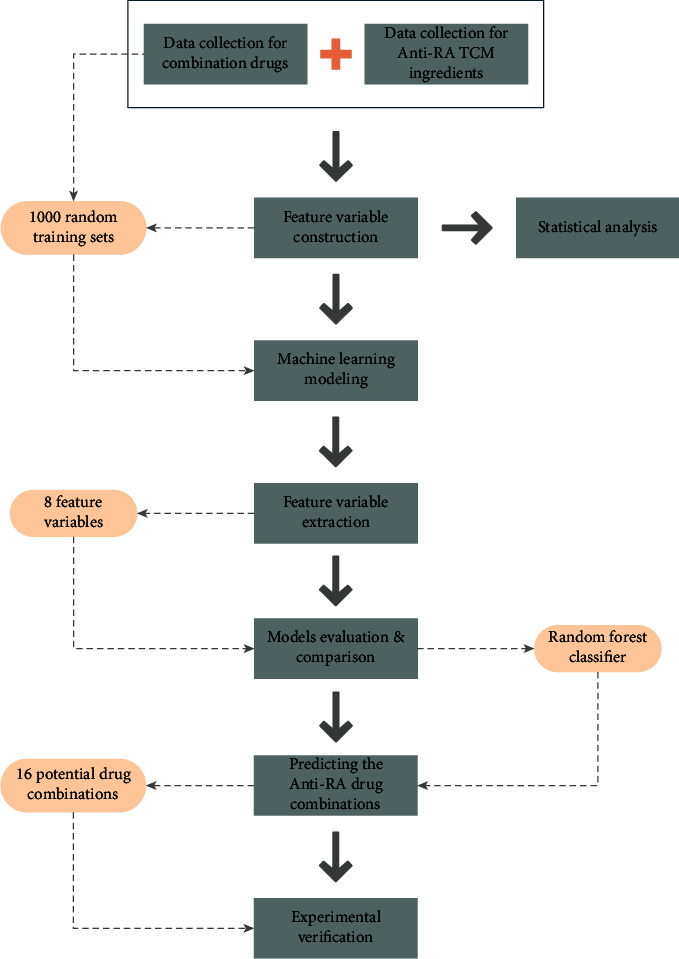
Flow chart of drug discovery and verification for anti-RA integrative medicine through machine learning modeling.

Therefore, we propose that the combination of Western medicine and TCM may be a promising method for improving the efficacy of anti-RA treatments and reducing side effects. Through their combination, the advantages of both Western medicine and TCM can be fully exploited to improve the therapeutic effects of RA treatment. However, because numerous active ingredients in TCM and hundreds of listed Western medicines have been reported to be effective in anti-RA treatments, the potential drug combinations are in the thousands or even tens of thousands. This multitude of combinations cannot feasibly be evaluated or verified through clinical practice and trials; therefore, the most practical method for identifying potential combinations would be to use drug data for existing combination drugs to model, evaluate, and screen potential anti-RA combinations of Western medicine and TCM and then experimentally validate these models. Therefore, in this study, we developed a classification and prediction model for combining Western and TCM drugs based on characteristics obtained through small molecule research on TCM and existing data on combined drugs. This model was developed using machine learning modeling followed by manual screening of anti-RA combination drugs. Through experimental verification, we identified effective combinations of Western and TCM drugs for treating RA; these findings can serve as a reference for clinical treatment of RA by using such combined treatment. The flow chart illustrating the process of drug discovery and verification for anti-RA integrative medicine through machine learning modeling is displayed in Figure 1.

## 2. Materials and Methods

### 2.1. Data Collection and Collation

The DrugCombDB database (http://drugcombdb.denglab.org/) is a comprehensive combinatorial drug database [15] containing integrated drug combinations from various data sources. To ensure the reliability of the research data, we first downloaded the Food and Drug Administration- (FDA-) provided known drug combination dataset from the database (up to May 31, 2019). The molecular formula, Chemical Abstracts Service (CAS) number, canonical simplified molecular-input line-entry system (SMILES), and other chemical informatics data corresponding to each combination of drugs and their action targets were collected from the DrugBank database (https://go.drugbank.com/).

Furthermore, we searched “Rheumatoid Arthritis” on the Traditional Chinese Medicine Systems Pharmacology (TCMSP) database (https://tcmspw.com/) to retrieve target Chinese medicine ingredient data reported to be relevant to treating RA. We obtained the main small molecules chemical information from the PubChem database (https://pubchem.ncbi.nlm.nih.gov/). In addition, several common anti-RA drugs were selected from the DrugBank database along with those discussed in the literature and those recommended by clinicians. All small molecule data for the TCM ingredients (collected from the TCMSP) and for known anti-RA Western drugs were combined in pairs to form the final prediction sample set.

### 2.2. Construction of Combined Drug Characteristic Variables

#### 2.2.1. Drug Combination Features Based on Molecular Fingerprint Similarity

Canonical SMILES of all drugs were collected from the DrugBank and PubChem databases, and the 1024-dimensional molecular fingerprints for each of the small molecule drugs were calculated using DRAGON software (version 7.0). The fingerprint similarity (*S*_FP_) between two small molecule drugs was then calculated using Tanimoto coefficient-based similarity:

(1) $$\begin{array}{c} S_{\text{FP}}\langle d_{A},d_{B}\rangle =\frac{A∙B}{{\left\|A\right\|}^{2}+{\left\|B\right\|}^{2}-A∙B}, \end{array}$$

where *A* is the 1024-dimensional molecular fingerprint feature vector corresponding to the small molecule drug *d*_*A*_, ‖*A*‖ is the vector length, *B* is the 1024-dimensional molecular fingerprint feature vector corresponding to the small molecule drug *d*_*B*_, ‖*B*‖ is the vector length, and *A*∙*B* is the vector inner product.

#### 2.2.2. Drug Combination Characteristics Based on Small Molecule ADME Similarity

To predict the adsorption, distribution, metabolism, and excretion (ADME) characteristics of each drug, Canonical SMILES for all small molecule drugs were imported into the SwissADME online system (http://www.swissadme.ch/) [16]. We selected 12 indicators as characteristic indexes for our ADME similarity calculations: molecular weight, heavy atoms, aromatic heavy atoms, fraction Csp3, rotatable bonds, H-bond acceptors, H-bond donors, molecular refractivity, topological polar surface area, Silicos-IT Log P, and Silicos-IT LogSw. All ADME indexes were first normalized, and the Euclidean distance was then used to calculate the similarity value *S*_ADME_ of ADME:

(2) $$\begin{array}{c} S_{\text{ADME}}={\left[\overset{n}{\underset{i=1}{\sum }}{\left(x_{i}^{A}-x_{i}^{B}\right)}^{2}\right]}^{1/2}, \end{array}$$

where *x*_*i*_^*A*^ is the ADME index corresponding to the drug small molecule *d*_*A*_ and *x*_*i*_^*B*^ is the ADME index corresponding to the drug small molecule *d*_*B*_ (*n* = 12).

#### 2.2.3. Drug Combination Characteristics Based on Sequence Similarity of Small Molecular Targets

The action targets of all drugs were obtained from DrugBank and the TCMSP, and the protr package (version 1.6.2) based on R language was used to obtain the sequence of action protein targets of all small molecule drugs. The sequence similarity between the two target proteins, *S*_Seq_〈*d*_*A*_, *d*_*B*_〉, was calculated using the Smith–Waterman algorithm. The maximum, minimum, median, and mean values of the sequence similarity *S*_Seq_〈*d*_*A*_, *d*_*B*_〉 between all target proteins of the *d*_*A*_ and *d*_*B*_ were included in the characteristic variables as *S*_Seqmax_, *S*_Seqmin_, *S*_Seqmed_, and *S*_Seqmean_, respectively.

#### 2.2.4. Drug Combination Characteristics Based on GO Functional Similarity of Small Molecular Targets

To calculate the gene ontology (GO) functional similarity *S*_GO_〈*d*_*A*_, *d*_*B*_〉 of the two drugs *d*_*A*_ and *d*_*B*_, we used the GOSemSim package (version 2.14.2). Similarly, the minimum, maximum, median, and mean values of the GO functional similarity *S*_GO_〈*d*_*A*_, *d*_*B*_〉 of the two drugs *d*_*A*_ and *d*_*B*_ were included in the characteristic variables as *S*_GOmin_, *S*_GOmax_, *S*_GOmed_, and *S*_GOmean_, respectively.

#### 2.2.5. Drug Combination Characteristics Based on Pathway Similarity of Small Molecule Targets

We used the clusterProfiler package (version 3.14.3) to perform a Kyoto Encyclopedia of Genes and Genomes (KEGG) Pathway enrichment analysis on all drug small molecule targets. We then screened the enrichment pathways of all targets based on *p* value < 0.05 and *q* value < 0.05. A vector, *P* = (*p*_1_, *p*_2_, ⋯, *p*_*n*_), consisting of all pathways was constructed according to the enriched pathways, {*p*_*k*_|*k* = 1, 2, ⋯, *n*} (where *n* is the number of pathways). If a target was enriched in a pathway *p*_*k*_ in the target set *T*_*A*_ of the drug small molecule *d*_*A*_, then the value was set as *p*_*k*_^*A*^ = 1 at the corresponding position of the drug small molecule *A* pathway vector *P*_*A*_, and if not, it was set as 0:

(3) $$\begin{array}{c} p_{k}^{A}=\left\{\begin{array}{cc} 1, & T_{A}\cap p_{k}\neq \emptyset ,\\ 0, & \text{others}. \end{array}\right. \end{array}$$

The target pathway vector *P*_*B*_ of the drug small molecule *d*_*B*_ can be similarly obtained using a string similarity formula to calculate the pathway similarity value (*S*_Pathway_) of two drugs:

(4) $$\begin{array}{c} S_{\text{Pathway}}\langle d_{A},d_{B}\rangle =\frac{P_{A}∙P_{B}}{\left\|P_{A}\right\|\times \left\|P_{B}\right\|}, \end{array}$$

where ‖*P*_*A*_‖ and ‖*P*_*B*_‖ are path vector lengths and *P*_*A*_ · *P*_*B*_ is the vector inner product.

#### 2.2.6. Drug Combination Characteristics Based on Distance of Small Molecule Targets in Human PPI Network

The distance and network proximity of small molecule targets of different combinations of drugs on the human protein–protein interaction (PPI) network were calculated to measure the synergy characteristics of combination drugs within the PPI network:

(5) $$\begin{array}{c} S_{\text{PPImin}}\langle d_{A},d_{B}\rangle =\frac{1}{1+e^{-\min\left(d\left(a,b\right)\right)}},\\ S_{\text{PPImax}}\langle d_{A},d_{B}\rangle =\frac{1}{1+e^{-\max\left(d\left(a,b\right)\right)}},\\ S_{\text{PPImed}}\langle d_{A},d_{B}\rangle =\frac{1}{1+e^{-\text{med}\left(d\left(a,b\right)\right)}},\\ S_{\text{PPImean}}\langle d_{A},d_{B}\rangle =\frac{1}{1+e^{-\langle d_{AB}\rangle }},\\ S_{\text{np}}\langle d_{A},d_{B}\rangle =S_{\text{PPImean}}\langle d_{A},d_{B}\rangle -\frac{S_{\text{PPImean}}\langle d_{A},d_{A}\rangle +S_{\text{PPImean}}\langle d_{B},d_{B}\rangle }{2}, \end{array}$$

where *d* (*a*, *b*) is the shortest path distance between two protein targets, *a* and *b*, within the PPI network (calculated using the igraph package [version 1.2.6]), 〈*d*_*AB*_〉 = 1/‖*A*‖ × ‖*B*‖∑_*a*∈*A*, *b*∈*B*_*d*(*a*, *b*), ‖*A*‖ is the number of targets for the drug small molecule *d*_*A*_, and ‖*B*‖ is the number of targets for the drug small molecule *d*_*B*_. The human PPI background network used in the calculation was derived from the literature [17]; the PPI network contained a total of 16677 proteins and 243603 interactions.

The Tanimoto coefficient-based similarity, Euclidean distance, and cosine similarity calculations used in this paper were calculated using the philentropy package (version 0.4.0) based on R language (version 4.0.2).

### 2.3. Construction and Selection of Combination Drug Prediction Model

To comprehensively determine and select an appropriate machine learning model for modeling and prediction in this study, we selected different machine learning classifiers for use with the training set and the verification set and compared their classification accuracies. In this study, we selected and compared five classical machine learning models: the *k*-nearest neighbors (*k*-NN) [18], naive Bayes (NB) [19], support vector machine (SVM) [20], random forest (RF) [21], and AdaBoost classifiers [22]. The caret package (version 6.0-86), the klaR package (version 0.6-15), the svmRadial model in the kernlab package (version 0.9-29), the randomForest package (version 4.6-14), and the ada package (version 2.0-5) were used for the *k*-NN, naive Bayes, support vector machine, random forest, and AdaBoost classifiers, respectively.

Furthermore, we used established sample training sets to compare the classification prediction effects of the five machine learning models. First, the sample set was divided into prediction training sets and internal validation sets at a 7 : 3 ratio. Then, the prediction training set was input into the five machine learning models, and the machine learning models were trained using three sets of 10-fold cross-validation. The trained machine learning models were evaluated using the internal validation sets, and the model with the best classification prediction effect was selected as the final drug prediction model for the Western and TCM drug combinations.

### 2.4. Evaluation of Machine Learning Models

In this study, we defined precision rate as the proportion of positive samples correctly predicted by the model out of all positive predictions, recall rate as the proportion of positive samples correctly predicted by the model out of all predictions, accuracy as the proportion of positive and negative samples to all samples correctly predicted by the model, and *F*1-score as the overall score of the model when precision rate and recall rate were given equal weight (calculated using Formulas (6)–(9), respectively). A higher score indicated a better classification effect.

(6) $$\begin{array}{c} \text{Precision}=\frac{\text{TP}}{\text{TP}+\text{FP}}, \end{array}$$

(7) $$\begin{array}{c} \text{Recall}=\frac{\text{TP}}{\text{TP}+\text{FN}}, \end{array}$$

(8) $$\begin{array}{c} \text{Accuracy}=\frac{\text{TP}+\text{TN}}{\text{TP}+\text{FN}+\text{FP}+\text{TN}}, \end{array}$$

(9) $$\begin{array}{c} F1‐\text{score}=2\times \frac{\text{Precision}\times \text{Recall}}{\text{Precision}+\text{Recall}}, \end{array}$$

where true positive (TP) indicates a sample is positive and was predicted to be positive, false negative (FN) indicates a sample is positive but was predicted to be negative, false positive (FP) indicates a sample is negative but was predicted to be positive, and true negative (TN) indicates a sample is negative and was predicted to be negative.

In the plot of the receiver operating characteristic (ROC) curve, the horizontal axis represents the false positive rate (FPR) and the vertical axis represents the true positive rate (TPR). The area under the ROC curve is the area surrounded by the ROC and its lower coordinate axis and is represented as AUROC in this paper. In addition, because the precision and recall rates of the model are not fixed, when the classification threshold of the model is adjusted, the precision and recall rates continue to change. The precision–recall curve represents the relationship between the precision rate of the ordinate and the recall rate of the abscissa with the change of the classification threshold *p* value; the area beneath this curve is represented as the area under the precision–recall curve (AUPRC).

### 2.5. *In Vitro* Cell Experiment Verification

#### 2.5.1. Cell Culture and Reagents

RAW264.7 cells were purchased from the American Type Culture Collection. The culture reagents included Dulbecco's modified Eagle medium (DMEM; Gibco, C11995500CP), Roswell Park Memorial Institute (RPMI) 1640 (Gibco, C11875500BT), fetal bovine serum (Bio IND, 04-002-1A), antimycotic (Lifetechnologies, 15240-112), phosphate-buffered saline (PBS), pH 7.4 (Gibco, 10010-023), trypsin-EDTA (0.05%; Lifetechnologies, 25300-054), bovine serum albumin (Lifetechnologies, 15561012), and CellTiter-Glo (CTG; PROMEGA, G7572). Consumables and instruments included a cell culture plate (Corning), cell culture flask (Corning), microplate tester (BioTek, HM-1), and conventional instruments, such as a CO_2_ incubator (Thermo 3111) and biosafety cabinet (Heal Force, HFSAFe-1200LC).

#### 2.5.2. Experimental Procedure and Methods

First, the different compounds were diluted with DMEM medium to the desired concentrations of the experimental design, namely, 0, 0.032, 0.16, 0.8, 4, 20, and 100 *μ*M. The cells were then counted and inoculated into 96-well culture plates at 2000 cells/100 *μ*L/well; 90 *μ*L of culture medium (including serum) was added to each well for overnight culture, and 10 *μ*L of drug solution was added. After a 48 h culture, CTG was used to detect the proliferation of RAW264.7 cells; CTG (100 *μ*L) was added to each well, the content of which was incubated at room temperature in a dark environment for 10 min. The chemiluminescence value of each well was measured at 500 ms using enzyme calibration.

#### 2.5.3. Combination Index Measurement

In this study, according to the principle of the median effect—specifically by employing the Chou–Talalay model [1]—the synergistic effect between each set of drugs was analyzed, and the effects of integrated Western and TCM drugs on RA cell lines were evaluated using the combination index (CI). The CI indicates the degree of drug synergy, addition, or antagonism for any given drug combination:

(10) $$\begin{array}{c} \text{CI}=\frac{D_{A}}{{\left(D_{A}\right)}_{x}}+\frac{D_{B}}{{\left(D_{B}\right)}_{x}}, \end{array}$$

where when CI = 1, the two drugs have an additive effect, when CI < 1, the two drugs have a synergistic effect, and when CI > 1, the two drugs are antagonistic. *D*_*A*_ and *D*_*B*_ represent the concentrations of the two drugs, *d*_*A*_ and *d*_*B*_, when administered in combination, and (*D*_*A*_)_*x*_ and (*D*_*B*_)_*x*_ represent the concentrations of the two drugs, *d*_*A*_ and *d*_*B*_, when they have been administered alone and the combined drug inhibition rate has been reached.

(11) $$\begin{array}{c} D_{x}=D_{m}{\left(\frac{f_{a}}{f_{u}}\right)}^{1/m}. \end{array}$$

In Formula (11), *f*_*a*_ and *f*_*u*_ are the inhibition and survival rates, respectively, when the two drugs are combined, *f*_*a*_ = 1 − *f*_*u*_, *D*_*m*_ is the median dose, and *m* is the curve coefficient (calculated according to half-maximal inhibitory concentration (IC_50_) theory).

In this study, CompuSyn software (http://www.combosyn.com/) was used to calculate the CI values of the drug combinations to evaluate whether each screened combination of Western and TCM drugs had synergistic therapeutic effects.

### 2.6. Statistical Analysis

Data were expressed as the mean ± standard deviation, and a *t*-test and Mann–Whitney nonparametric tests were performed using IBM SPSS Statistics (version 26.0).

## 3. Results

### 3.1. Collection and Collation of Combined Drug Data

We obtained 946 pairs (comprising 816 drugs) of combinatorial drug data reported by the FDA from the DrugCombDB database. We further downloaded all existing small molecule drug data from the DrugBank database. After name matching and sorting, some small molecule drug data without key information, such as targets, were removed. In total, we included 488 drugs and their corresponding 546 pairs of combinations. The data obtained for each small molecule drug mainly included DrugBank ID, canonical SMILES, and known targets (Supplementary 1; pretreated FDA-certified combination drug dataset).

### 3.2. Construction of Combination Drug Training Set

First, using the method described in Section 2.2, we obtained the molecular fingerprint similarity value (*S*_FP_), the ADME index similarity value (*S*_ADME_), and the minimum, maximum, median, and mean of the similarity (*S*_Seqmi*n*_, *S*_Seqmax_, *S*_Seqmed_, and *S*_Seqmean_, respectively) of the drug target sequence between the two small molecule drugs. The minimum, maximum, median, and mean values of the GO functional similarity of the drug targets were *S*_GOmin_, *S*_GOmax_, *S*_GOmed_, and *S*_GOmean_, respectively. The KEGG pathway similarity value (*S*_Pathway_); the minimum, maximum, median, and mean of the shortest path of different drug targets on the human PPI network (*S*_PPImin_, *S*_PPImax_, *S*_PPImed_, and S_PPImean_, respectively); and the proximity (*S*_np_) of the different drug targets were also obtained. Thus, we obtained a total of 16 characteristic variables to predict combinations of Western and TCM drugs.

In this study, we evaluated 16 characteristic variables between two drugs for 546 pairs of combined drugs and removed those for which characteristic variables could not be calculated or were missing. We obtained a total of 404 positive samples of combined drugs (comprising 488 drugs) reported by the FDA. Due to a lack of effective noncombination drug data, we combined the 488 drugs in pairs, removed the existing combination drug samples and those missing data, and obtained a total of 45603 noncombination drugs as negative samples (Supplementary 2; positive and negative sample training dataset for all drug combinations).

**Figure 2:**
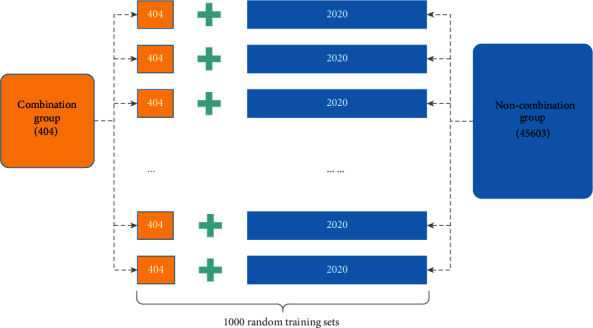
Training set of prediction samples comprised 1000 training sets of prediction samples randomly selected from 2020 samples of noncombination drugs and 404 samples of combination drugs.

Finally, to construct the training set for the TCM-combined drug samples, because the number of noncombined drugs should be much larger than that of combined drugs and to avoid large deviation in positive and negative samples, we set the ratio of positive to negative samples as 1 : 5; the number of positive samples was 404, and the number of negative samples randomly extracted from the 45603 noncombined drug samples was 404 × 5 = 2020. Through this, a training sample set was formed containing 2424 samples, and the training set was repeated 1000 times to randomly generate 1000 prediction training sets of combined drugs, as displayed in Figure 2.

### 3.3. Analysis and Screening of Characteristic Variables in Combination Drug Training Set

**Figure 3:**
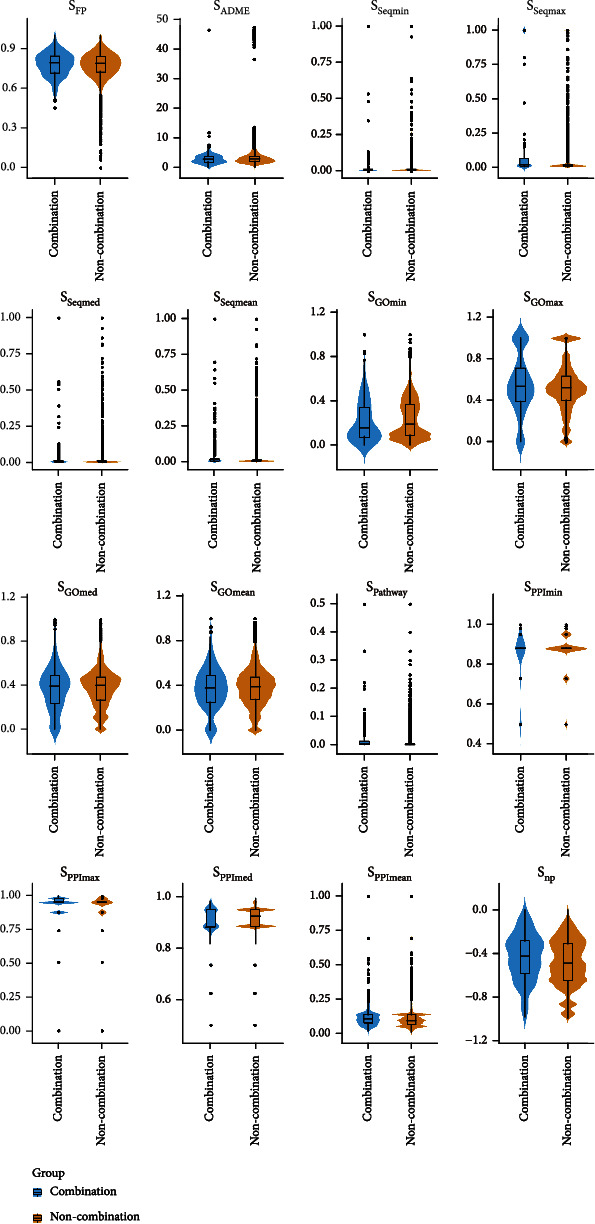
Violin diagram of value distribution of 16 characteristic variables in training sample set.

A *t*-test and a Mann–Whitney nonparametric test were used to determine whether significant differences existed between the 16 characteristic variables in the combined drug group and those in the noncombined drug group (*p* < 0.05). The results revealed that *S*_FP_, *S*_ADME_, *S*_Seqmin_, *S*_GOmax_, *S*_GOmed_, *S*_GOmean_, and *S*_PPImax_ did not significantly differ between the combined drug and noncombined drug groups (*p* > 0.05). Significant differences were found in the *S*_Seqmax_, *S*_Seqmed_, *S*_Seqmean_, *S*_Pathway_, *S*_PPImin_, *S*_PPImed_, *S*_PPImean_, and *S*_np_ between the two statistical tests (*p* < 0.05). Statistical differences were found in *S*_GOmin_ in the nonparametric tests (*p* < 0.05). All statistical tests were performed in IBM SPSS Statistics (version 26.0), and their data are presented in Table 1. The violin diagrams of the distribution of characteristic variables in different groups were completed using ggplot2 (version 3.3.5) and are displayed in Figure 3.

**Figure 4:**
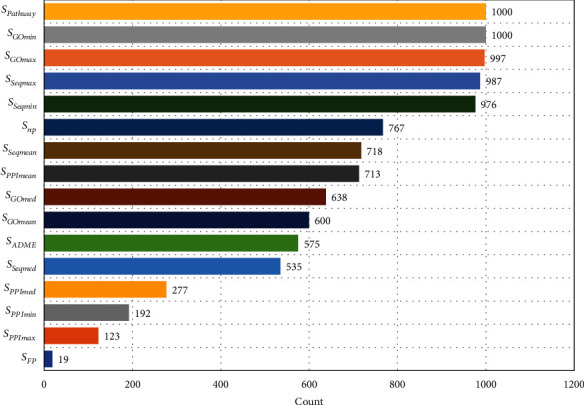
Occurrence of 16 feature variables from 1000 feature extractions.

An *rfe* function using the caret package was used to screen the 16 characteristic variables. A random forest model was used as the selection function, which was *functions* = *rfFuncs*, and the method was cross-validation, which was *method* =^“^*cv*.^”^ The others parameters were set to default. The training set was repeated 1000 times to extract feature variables from 1000 randomly generated training set samples, and each extracted feature variable set was recorded. The frequency of each feature variable in the 1000 feature selections was counted; the results are displayed in Figure 4.

As illustrated in Figure 4, eight characteristic variables, namely, *S*_Pathway_, *S*_GOmin_, *S*_GOmax_, *S*_Seqmin_, *S*_Seqmax_, *S*_Seqmean_, *S*_PPImean_, and *S*_np_, had appearance frequencies of over 70% after 1000 characteristic screenings. *S*_Pathway_ and *S*_GOmin_ were identified as key characteristic variables for predicting combined drugs. The other eight indicators had appearance frequencies below 70%; for example, *S*_FP_ had a rate of less than 10%. Thus, we removed the group of indicators with frequencies lower than 70% and included the top eight indicators as feature variables for subsequent machine learning.

### 3.4. Comparison and Selection Results of Machine Learning Models

**Figure 5:**
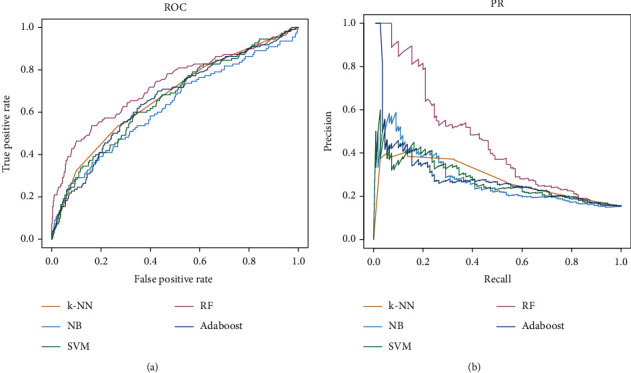
ROC and PR curves of five machine learning models for classification prediction.

The five included machine learning models were subjected to three replicates of 10-fold cross-validation under 1000 random samples to evaluate the predictive classification performance of different models. The results are displayed in Table 2, and the ROC and PR curves of the five models are presented in Figure 5.

Through evaluation and comparison of the predictive ability of the different models, we found that the classification index scores of the random forest model were generally better than those of the other prediction classifiers. Therefore, the random forest model was adopted as the combined drug prediction classifier for this study. The parameter was represented as *ntree* = 300.

### 3.5. Prediction Results of Integrative Medicine

We entered “Rheumatoid Arthritis” into the TCMSP database as a search term and identified 41 small molecule types in TCM ingredients that have been used to treat RA. We collected the target treatment information for each small molecule and verified it using the Uniprot database (https://www.uniprot.org/). We collected the molecular formula, PubChem CID, CAS number, and canonical SMILES data of each small molecule from PubChem (Table 3).

The information for drugs commonly used in RA treatment was also obtained from DrugBank. In this study, acetaminophen, celecoxib, choline salicylate, diclofenac, hydroxychloroquine, indometacin, leflunomide, methotrexate, naproxen, sulfasalazine, and 10 other common drugs were selected, as presented in Table 4.

The aforementioned 41 small molecule TCM (collected from TCMSP) and 10 common anti-RA Western medicine drugs were combined in pairs to obtain potential drug combinations: *C*_41_^1^ × *C*_10_^1^ = 410 combinations. Thus, we obtained the final prediction sample set (Supplementary 3; drug combination prediction dataset of integrated Western and TCM drugs).

The results revealed that out of 1000 random sample predictions, 34, 62, and 114 pairs of Western and TCM drugs were predicted to be combination drugs more than 500, 100–500, and 1–100 times, and 200 groups were predicted to be noncombination drugs. In this study, we considered predictions of more than 100 times to be potential anti-RA Western and TCM combination drugs, with higher numbers of positive sample predictions indicating a greater potential for drug combination. Therefore, we selected the 15 groups that were predicted more than 700 times for subsequent cell experiments. The combination drug information is displayed in Table 5 (Supplementary 4; prediction results of all Western and TCM combinations).

### 3.6. Experimental Verification Results

Because RAW264.7 cells were used for experimental verification, we first determined whether a single small molecule had an inhibitory effect on RAW264.7 cells. We then tested the Western and TCM drug combinations. Finally, the CI value was calculated using CompuSyn software to determine whether the small molecule combinations of the two drugs were synergetic.

**Figure 6:**
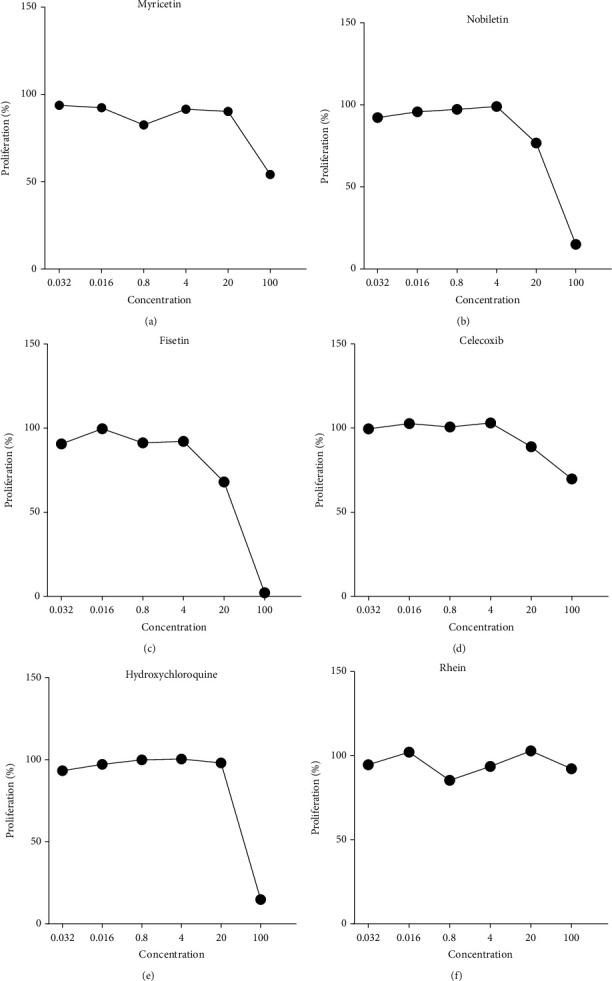
Effects of myricetin, nobiletin, fisetin, celecoxib, hydroxychloroquine, and rhein on the viability of RAW264.7 cells.

We then applied the method described in Section 2.5. Based on observations of the proliferation effect of the 15 small molecules on the RAW264.7 cells, myricetin, nobiletin, fisetin, celecoxib, and hydroxychloroquine significantly reduced the survival rate of the RAW264.7 cells. The IC_50_ values of the myricetin, nobiletin, fisetin, celecoxib, and hydroxychloroquine were> 100, 23.93, 31.19, >100, and 79.43 *μ*mol/L, respectively, as presented in Figure 6.

We further tested combinations of Western and TCM containing these five small molecules (a total of seven pairs) based on the prediction results of the machine learning model and calculated the CI values, as displayed in Table 6. We found that of the seven pairs of Western and TCM combinations, five of the pairs had CI values less than 0.7, indicating that these drugs have good synergy.

## 4. Discussion

Machine learning methods have been widely used in many fields of research, such as text classification [23], sentiment analysis [24], breast cancer diagnosis [25], web page classification [26], and image generation [27]. Many researchers have used computational and modeling methods to identify potential combinatorial drugs [17, 28–30], and the use of machine learning to predict drug combinations has notably increased in recent years [31–34]. However, nearly all of this research has focused of combinations of Western drugs. Combinations of Western and TCM drugs have rarely been reported, especially within the context of RA treatment. TCM is a complex system composed of many small molecules with synergistic effects. In recent years, the research of TCM small molecule is still the focus of modern pharmacology. We mainly focused on the prediction of the combination of small molecule with clear pharmacological effects in TCM and Western medicines that have been approved drugs. Therefore, in this study, we examined the small molecule characteristics of TCM ingredients that differed from those in Western combination drugs. We developed a method for complete modeling and prediction to screen anti-RA combination drugs.

For feature variable construction, we selected drug structures and ADME features. However, the results of the statistical analyses and feature variable screening revealed that drug structure similarity and ADME characteristics had no significant effect on the classification of combination drugs. Notably, sequence similarity, GO functional similarity, and the distance of drug targets within the PPI network of the small molecule drug targets were found to be related to the combination of drugs, with the similarity value of the drug target pathway (*S*_Pathway_) being identified as a key feature in combination drugs, both statistically and in feature screening. Therefore, the synergistic mechanism of two or more drugs within a pathway may serve as a key reference for future developments of combined drugs.

In constructing the sample training set, we were unable to collect sufficient noncombined drug data (namely the number of negative samples) due to limited research data. Therefore, in the original data, the number of positive samples was only 1% that of negative samples, which created a serious imbalance in the number of positive and negative samples. If the model had been constructed using the original datasets, the disproportionate number of negative samples would have caused overfitting, and the predictions would have been biased toward the classifications of the larger number of samples. This would have greatly reduced the normalizing ability of the models. However, simply reducing the sample size may have excluded potential drug combinations in the 45603 samples.

To effectively and reasonably overcome this challenge, we proposed a method in which multiple training sets were constructed using random sampling performed 1000 times. For each instance of modeling, the positive samples remained unchanged, and a limited number of negative samples were randomly selected from the total negative samples; 1000 training sets were randomly composed. The machine learning model was trained by and predicted the 1000 training sets. The final potential combination drugs were then determined on the basis of the percentage of each sample predicted to be a combination drug (positive) in the 1000 learning sessions. However, because the number of noncombination drugs is generally much larger than that of combination drugs but large deviations in numbers of positive and negative samples would cause prediction deviation in the machine learning model, in this study, the ratio of positive and negative samples was set to 1 : 5. Through these changes, we were able to prevent imperfect data sampling of combined drugs.

In this study, from the prediction and evaluation results of five different machine learning classification models in 1000 random sample training sets, the accuracy, precision, *F*1 score, AUROC, and AUPR values of RF are the highest among the five machine learning models. AdaBoost performs best on recall, followed by SVM and RF. The prediction scores of *k*-NN and NB on these six machine learning evaluation indexes were lower, suggesting that these two models may not be suitable for combination drug prediction. The prediction performance of SVM and AdaBoost is second only to that of RF. Therefore, through the evaluation and comparison of the predictive ability of different models, we found that the RF classification index scores are generally better than other predictive classifiers. Therefore, the RF model is used as the final combined drug prediction classifier, where the parameter *ntree* = 300. We used the RF classifier to predict 410 groups of Western and TCM drug combinations to treat RA and thus preliminarily predicted 96 pairs of potential Western and TCM drug combinations to treat RA. We used RAW264.7 cells to experimentally verify the effects of a single drug small molecule on the proliferation of RAW264.7 cells. We then verified the effects of combined drugs on the cells. The results revealed that five of the seven selected pairs of combined Western and TCM drugs had high synergistic effects, indicating that our model had high accuracy and practicability. The five optimal combinations were Rhein and Celecoxib, Nobiletin and Celecoxib, Myricetin and Celecoxib, Fisetin and Celecoxib, and Rhein and Hydroxychloroquine.

Notably, celecoxib was found to have a higher synergistic therapeutic effect when combined with several small molecule TCM ingredients, namely, myricetin, rhein, nobiletin, and fisetin. In addition, rhein did not demonstrate an inhibitory effect on RAW264.7 cell proliferation when applied alone. However, when combined with celecoxib (CI < 0.7), the combined inhibition rate of 100 *μ*mol/L rhein and 20 *μ*mol/L celecoxib was 35.68%, which was higher than the rate of each of the two alone. Rhien had high synergy with hydroxychloroquine.

Notably, triptolide combined with Western drugs, such as methotrexate, has previously been reported to produce favorable results in past experiments [35]. However, the number of predicted combinations containing triptolide in our model was not high; it predicted triptolide combined with diclofenac (75 times), naproxen (75 times), and celecoxib (24 times) but did not predict triptolide combined with methotrexate (0 times). This may be due to triptolide's reported clinical toxicity [36]. Although triptolide has been clinically reported to improve treatment outcomes, it may not be the best option for drug combinations and may even lead to more systemic side effects [37]. Our prediction results may support this hypothesis. The screening of potential combination of TCM and Western medicine based on the machine learning combined with experimental validation methods can improve efficiency and reduce research costs.

In conclusion, we constructed combination drug characteristics based on existing combination drug data and characteristics from TCM drug research. Key characteristic indexes were screened, and a random forest classification model was used to predict potential combinations of Western and TCM drugs for anti-RA treatment. Through experimental verification, we preliminarily demonstrated that this method of combination therapy for anti-RA treatment can be applied to clinical practice. In the future, we will conduct vivo experiments and clinical trials to validate the findings of this study. In addition, our machine learning model for integrated Western and TCM drug combination can be applied for predicting not only anti-RA drug combinations but also TCM combinations for other diseases.

## Figures and Tables

**Table 1:** Results of *t*-test and Mann–Whitney nonparametric test for 16 characteristic variables between different groups.

Feature	Noncombination group	Combination group	*t*-test	Mann–Whitney test
*S*_FP_	0.783 ± 0.087	0.783 ± 0.091	0.914	0.664
*S*_ADME_	3.347 ± 2.994	3.160 ± 2.697	0.167	0.199
*S*_Seqmin_	0.014 ± 0.045	0.020 ± 0.070	0.109	0.400
*S*_Seqmax_	0.066 ± 0.189	0.173 ± 0.342	∗∗	∗∗
*S*_Seqmed_	0.021 ± 0.057	0.030 ± 0.084	∗	∗∗
*S*_Seqmean_	0.026 ± 0.062	0.049 ± 0.105	∗∗	∗∗
*S*_GOmin_	0.236 ± 0.175	0.221 ± 0.193	0.114	∗∗
*S*_GOmax_	0.523 ± 0.239	0.550 ± 0.288	0.061	0.092
*S*_GOmed_	0.374 ± 0.172	0.369 ± 0.205	0.649	0.532
*S*_GOmean_	0.376 ± 0.166	0.374 ± 0.194	0.825	0.644
*S*_Pathway_	0.007 ± 0.029	0.014 ± 0.039	∗∗	∗∗
*S*_PPImin_	0.867 ± 0.092	0.821 ± 0.142	∗∗	∗∗
*S*_PPImax_	0.931 ± 0.116	0.934 ± 0.094	0.500	0.889
*S*_PPImed_	0.917 ± 0.043	0.910 ± 0.050	∗∗	∗∗
*S*_PPImean_	0.100 ± 0.064	0.121 ± 0.091	∗∗	∗∗
*S*_np_	−0.487 ± 0.215	−0.436 ± 0.216	∗∗	∗∗

∗*p* < 0.05 and^∗∗^*p* < 0.01.

**Table 2:** Prediction evaluation results of five machine learning classification models under 1000 random sample training sets.

Model	Accuracy	Precision	Recall	F1 score	AUROC	AUPR
*k*-NN	0.827 ± 0.012	0.848 ± 0.012	0.965 ± 0.013	0.903 ± 0.008	0.651 ± 0.024	0.309 ± 0.033
NB	0.813 ± 0.014	0.845 ± 0.012	0.949 ± 0.013	0.894 ± 0.009	0.617 ± 0.026	0.269 ± 0.031
SVM	0.834 ± 0.011	0.838 ± 0.012	0.992 ± 0.006	0.909 ± 0.007	0.657 ± 0.027	0.330 ± 0.038
RF	0.849 ± 0.012	0.861 ± 0.012	0.977 ± 0.008	0.915 ± 0.007	0.739 ± 0.024	0.460 ± 0.039
AdaBoost	0.833 ± 0.011	0.834 ± 0.012	0.998 ± 0.004	0.909 ± 0.007	0.667 ± 0.027	0.317 ± 0.038

**Table 3:** Small molecules of 41 anti-RA TCM ingredients collected from TCMSP database.

TCMSP ID	Name	Molecular formula	PubChem CID	CAS
MOL006505	(-)-Epicatechin	C15H14O6	72276	490-46-0
MOL006821	(-)-Epigallocatechin gallate	C22H18O11	65064	989-51-5
MOL008680	Acetaldehyde	C2H4O	177	75-07-0
MOL000475	Anethole	C10H12O	637563	104-46-1
MOL000008	Apigenin	C15H10O5	5280443	520-36-5
MOL002773	Beta-carotene	C40H56	5280489	7235-40-7
MOL000358	Beta-Sitosterol	C29H50O	222284	83-46-5
MOL003973	Caffeine	C8H10N4O2	2519	58-08-2
MOL008842	Chenodeoxycholic acid	C24H40O4	10133	474-25-9
MOL000390	Daidzein	C15H10O4	5281708	486-66-8
MOL000254	Eugenol	C10H12O2	3314	97-53-0
MOL013179	Fisetin	C15H10O6	5281614	528-48-3
MOL000392	Formononetin	C16H12O4	5280378	485-72-3
MOL000481	Genistein	C15H10O5	5280961	446-72-0
MOL002467	Gingerol	C17H26O4	442793	23513-14-6
MOL000666	Hexanal	C6H12O	6184	66-25-1
MOL005916	Irisolidone	C17H14O6	5281781	2345-17-7
MOL000422	Kaempferol	C15H10O6	5280863	520-18-3
MOL000305	Lauric acid	C12H24O2	3893	143-07-7
MOL000006	Luteolin	C15H10O6	5280445	491-70-3
MOL007990	Militarin	C34H46O17	171638	58139-23-4
MOL002008	Myricetin	C15H10O8	5281672	529-44-2
MOL003493	Naphthalene	C10H8	931	91-20-3
MOL003403	Nicotine	C10H14N2	89594	54-11-5
MOL005828	Nobiletin	C21H22O8	72344	478-01-3
MOL006214	Progesterone	C21H30O2	5994	57-83-0
MOL012297	Puerarin	C21H20O9	5281807	3681-99-0
MOL000098	Quercetin	C15H10O7	5280343	117-39-5
MOL012744	Resveratrol	C14H12O3	445154	501-36-0
MOL002268	Rhein	C15H8O6	10168	478-43-3
MOL011865	Rosmarinic acid	C18H16O8	5281792	20283-92-5
MOL006356	Sorbitol	C6H14O6	5780	50-70-4
MOL007154	Tanshinone IIA	C19H18O3	164676	568-72-9
MOL003186	Tripterine	C29H38O4	122724	34157-83-0
MOL003187	Triptolide	C20H24O6	107985	38748-32-2
MOL004932	Uralsaponin A	C42H62O16	128229	103000-77-7
MOL000511	Ursolic acid	C30H48O3	64945	77-52-1
MOL000635	Vanillin	C8H8O3	1183	121-33-5
MOL000173	Wogonin	C16H12O5	5281703	632-85-9
MOL009357	Yakuchinone A	C20H24O3	133145	78954-23-1
MOL009358	Yakuchinone B	C20H22O3	6440365	81840-57-5

**Table 4:** Information from DrugBank database for 10 common anti-RA small molecule drugs.

ID	Name	Molecular formula	PubChem CID	CAS
DB00316	Acetaminophen	C8H9NO2	1983	103-90-2
DB00328	Indometacin	C19H16ClNO4	3715	53-86-1
DB00482	Celecoxib	C17H14F3N3O2S	2662	169590-42-5
DB00563	Methotrexate	C20H22N8O5	126941	59-05-2
DB00586	Diclofenac	C14H11Cl2NO2	3033	15307-86-5
DB00788	Naproxen	C14H14O3	156391	22204-53-1
DB00795	Sulfasalazine	C18H14N4O5S	5339	599-79-1
DB01097	Leflunomide	C12H9F3N2O2	3899	75706-12-6
DB01611	Hydroxychloroquine	C18H26ClN3O	3652	118-42-3
DB14006	Choline salicylate	C12H19NO4	54686350	2016-36-6

**Table 5:** Combination data for 15 most predicted drug combinations.

Group	TCM ingredient	Anti-RA drug	Count
1	Eugenol	Acetaminophen	993
2	(-)-Epicatechin	Celecoxib	957
3	Rosmarinic acid	Celecoxib	946
4	Chenodeoxycholic acid	Acetaminophen	929
5	Eugenol	Diclofenac	912
6	Eugenol	Naproxen	910
7	Myricetin	Celecoxib	908
8	Chenodeoxycholic acid	Diclofenac	898
9	Chenodeoxycholic acid	Naproxen	897
10	Rhein	Celecoxib	894
11	Anethole	Naproxen	854
12	Anethole	Diclofenac	853
13	Nobiletin	Celecoxib	832
14	Fisetin	Celecoxib	791
15	Rhein	Hydroxychloroquine	770

**Table 6:** Effect of seven predicted pairs of Western and TCM combinations on RAW264.7 cell viability.

Combination	TCM (*μ*mol/L)	Drug (*μ*mol/L)	IC50	RAW264.7 cell inhibition rate (%)	CI
Rhein+celecoxib			>100 + 100		CI < 1
	100	100		39.31 ± 2.92	CI = 0.37
	20	100		35.68 ± 1.49	CI = 0.49
	4	100		38.60 ± 0.80	CI = 0.39
	100	20		20.08 ± 1.22	CI = 0.40
	100	4		14.22 ± 1.59	CI = 0.17
	20	20		20.17 ± 1.49	CI = 0.40

Nobiletin+celecoxib			8.83 + 100		CI < 1
	100	100		96.20 ± 0.24	CI = 0.01
	100	20		61.50 ± 1.67	CI = 0.17

Myricetin+celecoxib			100 + 4		CI < 1
	100	100		64.05 ± 0.94	CI = 0.06
	20	100		37.13 ± 2.13	CI = 0.46
	4	100		37.96 ± 1.58	CI = 0.41
	100	20		53.92 ± 1.59	CI = 0.03
	100	4		49.97 ± 0.93	CI = 0.02

Fisetin+celecoxib			10.89 + 100		CI < 1
	100	100		99.06 ± 0.03	CI = 0.02
	4	100		57.80 ± 1.89	CI = 0.88

Rhein+hydroxychloroquine			100 + 57.15		CI < 1
	100	20		86.88 ± 0.04	CI = 0.28

Epicatechin+celecoxib			>100 + 100		CI > 1

Rosmarinic acid+celecoxib			>100 + 100		CI > 1

## Data Availability

The data used to support the findings of this study are included within the article.

## Abbreviations

ADME: Adsorption, distribution, metabolism, and excretion
AUPRC: Area under the precision–recall curve
CAS: Chemical Abstracts Service
CI: Combination index
DMARDs: Disease modifying antirheumatic drugs
FDA: Food and Drug Administration
FN: False negative
FP: False positive
FPR: False positive rate
GO: Gene ontology
KEGG: Kyoto Encyclopedia of Genes and Genomes
*k*-NN: *k*-nearest neighbors
NB: Naive Bayes
NSAIDs: Nonsteroidal anti-inflammatory drugs
PPI: Protein–protein interaction
RA: Rheumatoid arthritis
RF: Random forest
ROC: Receiver operating characteristic
SMILES: Canonical simplified molecular-input line-entry system
SVM: Support vector machine
TCM: Traditional Chinese medicine
TCMSP: Traditional Chinese Medicine Systems Pharmacology
TN: True negative
TP: True positive
TPR: True positive rate.

## Supplementary Materials

Supplementary 1 Pretreated FDA-certified combination drug dataset.

Supplementary 2 Positive and negative sample training dataset for all drug combinations.

Supplementary 3 Drug combination prediction dataset of integrated Western and TCM drugs.

Supplementary 4 Prediction results of all Western and TCM combinations.
